# Antimicrobial Resistance and Biofilm Formation in *Enterococcus* spp. Isolated from Humans and Turkeys in Poland

**DOI:** 10.1089/mdr.2018.0221

**Published:** 2019-03-08

**Authors:** Anna Woźniak-Biel, Gabriela Bugla-Płoskońska, Jakub Burdzy, Kamila Korzekwa, Sebastian Ploch, Alina Wieliczko

**Affiliations:** ^1^ Department of Epizootiology with Clinic of Birds and Exotic Animals, Wroclaw University of Environmental and Life Sciences, Wroclaw, Poland.; ^2^ Department of Microbiology, Institute of Genetics and Microbiology, University of Wroclaw, Wroclaw, Poland.; ^3^ IT Lab, Faculty of Veterinary Medicine, Wroclaw University of Environmental and Life Sciences, Wroclaw, Poland.

**Keywords:** *Enterococcus*, resistance genes, multidrug resistance, biofilm

## Abstract

Enterococci are a natural component of the intestinal flora of many organisms, including humans and birds. As opportunistic pathogens, they can cause fatal infections of the urinary tract and endocarditis in humans, whereas in poultry symptoms are joint disease, sepsis, and falls in the first week of life. The study covered 107 *Enterococcus* strains—56 isolated from humans and 51 from turkeys. Among the isolates investigated *Enterococcus faecalis* was detected in 80.36% of human and 80.39% of turkey samples. *Enterococcus faecium* was identified in 8.93% of human and 17.65% of turkey strains. The highest percentage of the strains was resistant to tetracycline as follows: 48 (85.71%) and 48 (94.12%) of human and turkey strains, respectively. Resistance to erythromycin occurred in 37.50% of the human and in 76.47% of turkey strains, otherwise 27.10% of all strains showed resistance to ciprofloxacin. Our study revealed that 25% of human and 15.69% of turkey strains were resistant to vancomycin. Multidrug resistance showed in 32.14% and 43.14% of human and turkey strains, respectively. The tetracycline resistance gene, *tetM,* was detected in 82.24% of all strains analyzed, whereas the *tetO* gene was found in 53.57% of human but only in 7.84% of turkey strains. The vancomycin resistance gene (*vanA*) was detected in seven *Enterococcus* strains (six isolated from turkeys and one from humans). The *ermB* gene (resistance to macrolide) was detected in 55.14% of all isolates (42.86% of human and 68.63% of turkey strains), whereas the *ermA* gene was detected in 17.65% of turkey but only in 3.57% of human isolates. All the strains had the ability to form biofilms. A stronger biofilm was formed after 24-hour incubation by strains isolated from turkeys, whereas after 48 hours of incubation all examined strains produced strong biofilm.

## Introduction

Enterococci are part of the normal intestinal flora of mammals, birds, and humans. Among all the enterococcal species, *Enterococcus faecalis* and *Enterococcus faecium* are the most commonly identified in human samples, whereas *Enterococcus gallinarum* and *Enterococcus casseliflavus* are less represented.^1^ Enterococci are the cause of nosocomial infections, most frequently associated with urinary tract infections, endocarditis, intra-abdominal and pelvic infections, catheter infections, surgical infections, and central nervous system infections.^2^ In poultry, *Enterococcus* infection is mainly associated with endocarditis in chickens, hepatic granulomas in turkeys, ascites in hens, and arthritis, osteomyelitis, or pulmonary hypertension in broilers.^3–5^

The overuse of antimicrobials, in human and veterinary medicine, can select resistant strains.^6^ Enterococci are possible vectors in dissemination of resistance genes inside or outside the genus, for example, to the resident bacteria during their passage through the gut.^7,8^ The occurrence of animal enterococci resistant to different antimicrobial agents represents a high risk for transmission of these bacteria to humans.^9–12^ According to the European Center for Disease Prevention and Control,^13^ infections caused by *E. faecalis* are a clinical problem in many European countries especially because of their resistance to aminoglycosides and vancomycin. Moreover, in the second half of the 20th century, in Europe, avoparcin (glycopeptide antibiotic) was widely used as a feed additive to promote growth and feed utilization in pigs and poultry. The use of avoparcin as a feed additive, banned in Europe in 1997, played an important role in selection of vancomycin-resistant strains.^6,14^

Regarding molecular mechanism explaining the resistance of *Enterococcus* spp. to different groups of antimicrobials, several genes were identified (*blaZ, vanA, vanB, vanC-1, tetK, tetO, tetM, ermA, ermB, ermC*, and *aac(6’)Ie-aph(2”)Ia*. The main mechanisms of antibiotic resistance in enterococci are well known and are widely described in the literature.^7,15–19^

Bacterial biofilm formation is an integral part of many diseases, both in humans and animals. It is estimated that >60% of bacterial infections are associated with the phenomenon of biofilm formation.^20^ One of the most important among the microbial virulence factors is the ability of the microorganism to form biofilms, because it facilitates adaptation to harsh environmental conditions.^21^ A biofilm is a complex community of microorganisms pervasive in the natural environment. The current conceptual model of a biofilm portrays an ingeniously complicated multispecies entity in which ecological microniches are created and occupied by specific organisms.^22^ Naturally occurring microbial biofilms that contain not only bacteria but also yeasts, fungi, algae, and protozoa may be present. An organization of multispecies bacterial consortia may enable coexistence of species that would otherwise outcompete each other and facilitate synergistic interactions and gene transfer.^23^

Enterococci able to form biofilm are an etiological factor for many infections, mostly wound, respiratory, and urinary tract infections, and have many novel features such as greater virulence and higher resistance to bactericides than in planktonic cells. This makes it hard to destroy the structure of the biofilm and eradicate it.^21^ A large capacity for adhesion in biofilms and forms of tolerance to unfavorable environmental conditions greater than in planktonic bacteria is the cause of contamination in the food industry, for example, in brewing.

Another problem in the treatment of such infections is the fact that biofilms are often resistant to antimicrobial drugs.^24,25^ Enterococcal biofilm is of significant interest because it protects bacteria against antimicrobials and host immunity and therefore is difficult to eradicate. Inhibition of biofilm synthesis by uropathogenic bacteria reduces the risk of infection development in the urinary tract.^26^

The aim of this study was to determine the antimicrobial susceptibility and genetic mechanism of resistance in strains collected from humans and turkeys, as well the ability of enterococci to form biofilm. To our knowledge this is the first report about antimicrobial resistance and ability to form biofilm by *Enterococcus* strains isolated from humans and turkeys in Poland.

## Materials and Methods

### Samples and bacteria

A total of 107 fecal samples were collected from humans (*n* = 56) and turkeys (*n* = 51), in 2015. Samples from humans were obtained from the Diagnostic Laboratory “Dialab” (Wroclaw, Lower Silesia, Poland), whereas turkey samples were collected from nine commercial turkey farms. On each farm, indicated as: Z, JK, JB, JR, R, M, MS, JM, and JZ, only a few turkey houses were located (from 3 to 7). Only one strain of *Enterococcus* spp. from one turkey house was isolated. Overall, 51 turkey flocks were sampled (Table 1).

**Table 1.** Species Identification of *Enterococcus* spp. Isolated from Humans (*n* = 56) and Turkeys (*n* = 51) Using Multiplex PCR

*Species*	*Human strains (*n* = 56)*		*Turkey strains (*n* = 51)*	
	n *(%)*	*No. of isolates*	n *(%)*	*No. of isolates*
*Enterococcus faecalis*	45 (80.36)	25, 35, 39, 44, 45, 80, 81, 90, 103, 121, 123, 125, 130, 131, 145, 150, 171, 173, 180, 184, 190, 191, 192, 196, 979, 982, 989, 1003, 1010, 1013, 1021, 1023, 1030, 1031, 1034, 1035, 1841, 1931, 1983, 2000, 2002, 133A, 133B, 179A, 179B	41 (80.39)	Z1–3
				JK1, JK3, JK4
				JB1–3, JB6, JB7
				JR1, JR2–7
				R1–4
				M2–4
				MS1–7
				JM3–7
				JZ1–4
*Enterococcus faecium*	5 (8.93)	172, 178, 1872, 1949, 2006	9 (17.65)	JK2
				JB4, JB5
				M1, M5-M7
				JM1, JM2
*Enterococcus gallinarum*	6 (10.71)	73, 77, 83, 1868, 1873, 1963	1 (1.96)	JZ5

The swabs were plated on chromogenic media Enterococcosel Agar (BD, Heidelberg, Germany) using a reduction seed method. The plates were then incubated for 24 hours at 37°C. Colonies that were found to be black in color were considered to be of the genus *Enterococcus* and subjected to further analyses. After incubation, colonies with typical enterococcal morphology were identified by Gram's staining and confirmed by PCR.

### DNA extraction

Genetic material was isolated using Genomic Mini (A&A Biotechnology, Gdańsk, Poland) according to the manufacturer's recommendations. The DNA was quantified spectrophotometrically (BioPhotometer; Eppendorf, Hamburg, Germany) and stored at −20°C.

### Multiplex PCR

A single PCR method was used to identify genus *Enterococcus*, whereas the species level of *E. faecalis, E. faecium*, or *E. gallinarum* was determined in multiplex PCR. The primer sequences, specific for simultaneous amplification of the *ddl* gene (*E. faecalis* and *E. faecium*) and *sodA* gene (*E. gallinarum*), are listed in Table 2. The protocols of the single and multiplex PCR were described by Ke *et al.*^27^ and Yean *et al.*,^29^ respectively. Strains used as positive controls were as follows: *E. faecalis* ATCC 51299, *E. faecium* ATCC 700221, and *E. gallinarum* ATCC 700425. Products obtained by amplification were divided by electrophoresis in 2% agarose gels. DNA bands were stained with Midori Green DNA Stain (Nippon Genetics Europe GmbH, Dueren, Germany) and visualized with an ultraviolet transilluminator.

**Table 2.** Primer Sequences Used for *Enterococcus* Species Identification

*Species*	*Gene*	*Sequence (5′→3′)*	*Amplicon (bp)*	*References*
*Enterococcus* spp.	*tuf*	TAC TGA CAA ACC ATT CAT GAT G	112	Ke *et al.*^27^
		AAC TTC GTC ACC AAC GCG AAC		
*E. faecalis*	*ddl*	GGC CCT CTT TTA TCT GAA CGA	734	Dutka-Malen *et al.*,^28^ Yean *et al.*^29^
		GCG ACT TAA GCC ACT TCC AT		
*E. faecium*	*ddl*	CGC AGA GCA TGA AGT GTC CA	557	Dutka-Malen *et al.*,^28^ Yean *et al.*^29^
		CTT CTC GGT TTT CTG CTT TTG TA		
*E. gallinarum*	*sodA*	TTA CTT GCT GAT TTT GAT TCG	190	Layton *et al.*^30^
		TGA ATT CTT CTT TGA AAT CAG		

### Antimicrobial susceptibility testing

Antimicrobial susceptibility was tested by the disk diffusion method on Mueller–Hinton agar (Oxoid, Hampshire, United Kingdom), following the Clinical and Laboratory Standards Institute guidelines.^31^ The criteria for selection of antimicrobials were based on the CLSI recommendation for *Enterococcus* spp. and on their practical significance for clinical use. The susceptibility to ampicillin (10 μg), amoxicillin/clavulanic acid (20/10 μg), vancomycin (30 μg), ciprofloxacin (5 μg), tetracycline (30 μg), erythromycin (15 μg), and gentamicin (120 μg) was studied for both *Enterococcus* spp. isolated from humans and turkeys. Because the activity of gentamicin against enterococci is not great, only a high-level aminoglycoside screening test was performed. Assays were repeated twice, each in duplicate, to confirm the reproducibility of the disk diffusion data. All antimicrobial susceptibility disks were supplied by Oxoid. Multidrug resistance (MDR) was considered when the isolates were resistant to three or more antimicrobials of different families.

### Antibiotic resistance genes

Vancomycin resistance genes (vanA, vanB, and vanC-1) were tested by PCR in all enterococcal isolates obtained from humans and turkeys. Resistance genes for other antibiotics, including tet*K*, tet*M*, tet*O*, erm*A*, erm*B*, erm*C*, *aac(6’)Ie-aph(2”)Ia*, and *blaZ*, were also tested by PCR. All primers used in this study are summarized in Table 3. To amplify the genes *vanA, vanB, vanC-1, aac(6’)Ie-aph(2”)Ia*, and *blaZ* single PCR was used. For detecting the presence of genes tet*K*, tet*M*, tet*O*, as well as erm*A*, erm*B*, erm*C*, multiplex PCR was used according to protocols described by Ng *et al.*^36^ and Sutcliffe *et al.*^37^

**Table 3.** Primers Used for PCR Detection of Antimicrobial Resistance Genes

*Gene*	*Primers*	*Sequence (5′→3′)*	*Amplicon (bp)*	*References*
*β-lactams*				
*blaZ*	blaZ-I	ACT TCA ACA CCT GCT GCT TTC	173	Martineau *et al.*^32^
	blaZ-II	TGA CCA CTT TTA TCA GCA ACC		
*Vancomycin*				
*vanA*	vanA-I	TCT GCA ATA GAG ATA GCC GC	377	Klare *et al.*^33^
	vanA-II	GG AGT AGC TAT CCC AGC ATT		
*vanB*	vanB-I	GCT CCG CAG CCT GCA TGG ACA	529	Fraimow *et al.*^34^
	vanB-II	ACG ATG CCG CCA TCC TCC TGA		
*vanC-1*	vanC-I	GAA AGA CAA CAG GAA GAC CGC	796	Clark *et al.*^35^
	vanC-II	ATC GCA TCA CAA GCA CCA ATC		
*Tetracycline*				
*tetK*	tetK-I	TCG ATA GGA ACA GCA GTA	169	Ng *et al.*^36^
	tetK-II	CAG CAG ATC CTA CTC CTT		
*tetM*	tetM-I	GTG GAC AAA GGT ACA ACG AG	406	
	tetM-II	CGG TAA AGT TCG TCA CAC AC		
*tetO*	tetO-I	AAC TTA GGC ATT CTG GCT CAC	515	
	tetO-II	TCC CAC TGT TCC ATA TCG TCA		
*Erythromycin*				
*ermA*	ermA-I	TCT AAA AAG CAT GTA AAA GAA	645	Sutcliffe *et al.*^37^
	ermA-II	CTT CGA TAG TTT ATT AAT ATT AGT		
*ermB*	ermB-I	GAA AAG GTA CTC AAC CAA ATA	639	
	ermB-II	AGT AAC GGT ACT TAA ATT GTT TAC		
*ermC*	ermC-I	TCA AAA CAT AAT ATA GAT AAA	642	
	ermC-II	GCT AAT ATT GTT TAA ATC GTC AAT		
*Aminoglycosides*				
*aac(6′)Ie-aph(2′′)Ia*	aac_aph-I	GAG CAA TAA GGG CAT ACC AAA AAT C	480	Kao *et al.*^38^
	aac_aph-II	CCG TGC ATT TGT CTT AAA AAA CTG G		

### Biofilm formation

Biofilm formation was examined using the method of O'Toole and Kolter^39,40^ with a few modifications. One microliter of a late-log-phase culture (1–2 × 10^8^ colony-forming units/mL) was added to 99 μL LB broth, Lennox (Merck, Darmstadt, Germany) in a 96-well microtiter plate, incubated for 24 and 48 hours at 37°C, and rinsed thoroughly with water to remove nonadherent cells. Crystal violet (125 μL, 1% [wt/vol]) was added to each well, incubated for 15 minutes, and rinsed thoroughly with water. To solubilize the crystal violet, 200 μL of 95% ethanol was added to each well and mixed. A 100 μL aliquot was removed to a new well and the absorbance was read at 595 nm (Infinite 200 PRO; Tecan, Switzerland). The cut-off value (ODc) was defined according to Stepanović *et al.*^41^ At least three replicates were performed for each strain. In this research the ODc value was 0.054. *E. faecalis* strains were classified as follows: OD ≤ ODc, no biofilm producer; ODc < OD ≤2 ODc, weak biofilm producer; 2 ODc < OD ≤4 ODc, medium biofilm producer; OD >4 ODc, strong biofilm producer. In 24 and 48 hours bacterial cultures the diverse effect on biofilm formation was recorded.

### Statistical analysis

Statistical analysis was performed by nonparametric Mann–Whitney *U* test using the PQStat Statistical Program version 1.6.2 (PQStat, Poznań, Poland). The ability to biofilm formation by human and turkey strains after 24 and 48 hours of incubation was statistically analyzed. A value of *p* ≤ 0.05 was considered statistically significant.

## Results

In total, 107 *Enterococcus* strains were collected from humans (*n* = 56) and from turkeys (*n* = 51). Among the human strains 45 (80.36%) were identified as *E. faecalis*, 5 (8.93%) as *E. faecium*, and 6 (10.71%) as *E. gallinarum.* The numbers of *Enterococcus* spp. isolated from turkeys were as follows: 41 (80.39%) *E. faecalis* strains, 9 (17.65%) *E. faecium*, and 1 (1.96%) *E. gallinarum* strain (Table 1).

### Antimicrobial susceptibility

The results of the resistance of *Enterococcus* strains to selected antimicrobials are given in Table 4. The highest percentage of the strains isolated from humans was resistant to tetracycline (48; 85.71%) and erythromycin (21; 37.5%). Moreover, 17 (30.36%) and 14 (25%) human strains were resistant to ciprofloxacin and vancomycin, respectively.

**Table 4.** Antimicrobial Susceptibility of *Enterococcus* Strains Isolated from Humans (*n* = 56) and Turkey (*n* = 51)

*Strains*	*Antimicrobials*^a^																	
	*AMP*			*AMX*			*VAN*			*CIP*			*TET*			*ERY*		
	*S*	*I*	*R*	*S*	*I*	*R*	*S*	*I*	*R*	*S*	*I*	*R*	*S*	*I*	*R*	*S*	*I*	*R*
Human strains	*n* (%)																	
*E. faecalis (n* = *45)*	38 (84.44)	0	7 (15.56)	39 (86.67)	1 (2.22)	5 (11.11)	21 (46.67)	16 (35.55)	8 (17.78)	10 (22.22)	21 (46.67)	14 (31.11)	5 (11.11)	2 (4.44)	38 (84.45)	6 (13.33)	21 (46.67)	18 (40)
*E. faecium (n* = *5)*	5 (100)	0	0	5 (100)	0	0	2 (40)	2 (40)	1 (20)	2 (40)	2 (40)	1 (20)	1 (20)	0	4 (80)	0	3 (60)	2 (40)
*E. gallinarum (n* = *6)*	5 (83.33)	0	1 (16.67)	6 (100)	0	0	0	1 (16.67)	5 (83.33)	0	4 (66.67)	2 (33.33)	0	0	6 (100)	0	5 (83.33)	1 (16.67)
Total (*n* = 56)	48 (85.71)	0	8 (14.29)	50 (89.28)	1 (1.79)	5 (8.93)	23 (41.07)	19 (33.93)	14 (25)	12 (21.43)	27 (48.21)	17 (30.36)	5 (8.93)	3 (5.36)	48 (85.71)	6 (10.71)	29 (51.79)	21 (37.50)
Turkey strains																		
*E. faecalis (n* = *41)*	32 (78.05)	1 (2.44)	8 (19.51)	32 (78.05)	2 (4.88)	7 (17.07)	22 (53.66)	13 (31.71)	6 (14.63)	19 (46.34)	17 (41.46)	5 (12.20)	3 (7.32)	0	38 (92.68)	5 (12.20)	7 (17.07)	29 (70.73)
*E. faecium (n* = *9)*	2 (22.22)	0	7 (77.78)	2 (22.22)	1 (11.11)	6 (66.67)	3 (33.33)	4 (44.45)	2 (22.22)	0	2 (22.22)	7 (77.78)	0	0	9 (100)	0	0	9 (100)
*E. gallinarum (n* = *1)*	0	0	1 (100)	0	0	1 (100)	0	1 (100)	0	1 (100)	0	0	0	0	1 (100)	0	0	1 (100)
Total (*n* = 51)	34 (66.67)	1 (1.96)	16 (31.37)	34 (66.67)	3 (5.88)	14 (27.45)	25 (49.02)	18 (35.29)	8 (15.69)	20 (39.22)	19 (37.25)	12 (23.53)	3 (5.88)	0	48 (94.12)	5 (9.8)	7 (13.73)	39 (76.47)

^a^ All examined human and turkey strains were susceptible to gentamicin (120 μg); results not presented in the table.

S, susceptible; I, intermediate; R, resistant; the specific values used for disk diffusion test for *Enterococcus* spp. are described in the Clinical and Laboratory Standards Institute Supplement.^31^

AMP, ampicillin (10 μg); AMX, amoxicillin/clavulanic acid (20/10 μg); VAN, vancomycin (30 μg); CIP, ciprofloxacin (5 μg); TET, tetracycline (30 μg); ERY, erythromycin (15 μg).

Among turkey strains, the highest percentage of the isolates was resistant to tetracycline (48; 94.12%) and erythromycin (39; 76.47%). In addition, 23.53% and 15.69% of turkey strains were resistant to ciprofloxacin and vancomycin, respectively.

All strains investigated, isolated from humans and turkeys, were susceptible to gentamicin. The percentage of strains resistant to ampicillin and amoxicillin/clavulanic acid obtained from turkeys was more than two times higher than *Enterococcus* strains obtained from humans.

MDR to three or more classes of antimicrobial agents was found among isolates obtained from humans (18; 32.14%), as well those obtained from turkeys (22; 43.14%) (Table 5). Among the turkey strains, four isolates (7.84%) were resistant to six different antimicrobial agents, whereas among human strains only one (1.79%) isolate was found to be resistant to six different antimicrobials.

**Table 5.** Multidrug Resistance Phenotypes in *Enterococcus* Strains Isolated from Humans and Turkeys

*Multidrug resistance profile*	*Human strains (*n* = 18)*			*Turkey strains (*n* = 22)*		
	*No. of strains*			*No. of strains*		
	E. faecalis	E. faecium	E. gallinarum	E. faecalis	E. faecium	E. gallinarum
AMP + AUG + VAN + CIP + TET + ERY	125			JB2, JB3	M5, M7	
AMP + AUG + CIP + TET + ERY	130, 133A, 133B				JB5, M6, JM2	
AMP + AUG + VAN + TET + ERY				JM7		
AMP + AUG + VAN + CIP + TET	39					
AMP + AUG + TET + ERY				JB6, JB7, JM6	JB4	JZ5
AMP + CIP + TET + ERY	45, 150			M3	M1	
AMP + VAN + CIP + TET			73			
AMP + AUG + ERY				JZ4		
CIP + TET + ERY	80, 123, 191, 1021			Z1, JB1,	JK2	
VAN + CIP + TET	25, 35					
VAN + TET + ERY	1931	1872	1868, 1873	JK3, M4, MS6		
Total *n* (%)	14 (77.78)	1 (5.55)	3 (16.67)	13 (59.09)	8 (36.36)	1 (4.55)

AUG, amoxicillin/clavulanic acid (20/10 μg).

### Antibiotic resistance genes

The presence of the *tetM* gene was found in 48 (85.71%) *Enterococcus* strains isolated from humans, whereas the genes *tetO* and *ermB* were found in 30 (53.57%) and 24 (42.86%) *Enterococcus* isolates, respectively (Table 6).

**Table 6.** Presence of Resistant Genes in *Enterococcus* spp. Isolated from Humans (*n* = 56) and Turkeys (*n* = 51)

*Isolates*	*Resistant genes,* n *(%)*										
	blaZ	vanA	vanB	vanC-1	tetK	tetO	tetM	ermA	ermB	ermC	aac(6’)Ie-aph(2”)Ia
Human isolates											
*E. faecalis* (*n* = 45)	0	0	0	1 (2.22)	0	21 (46.67)	38 (84.44)	2 (4.44)	19 (42.22)	0	13 (28.89)
*E. faecium* (*n* = 5)	0	0	0	0	0	5 (100.00)	4 (80.00)	0	2 (40.00)	0	0
*E. gallinarum* (*n* = 6)	0	1 (16.67)	0	6 (100.00)	0	4 (66.67)	6 (100.00)	0	3 (50.00)	0	0
Total (*n* = 56)	0	1 (1.79)	0	7 (12.50)	0	30 (53.57)	48 (85.71)	2 (3.57)	24 (42.86)	0	13 (23.21)
Turkey isolates											
*E. faecalis* (*n* = 41)	2 (4.88)	5 (12.19)	0	0	0	3 (7.32)	32 (78.05)	5 (12.19)	27 (65.85)	0	4 (9.76)
*E. faecium* (*n* = 9)	0	1 (11.11)	0	0	0	1 (11.11)	7 (77.78)	3 (33.33)	7 (77.78)	0	4 (44.44)
*E. gallinarum* (*n* = 1)	0	0	0	1 (100.00)	0	0	1 (100.00)	1 (100.00)	1 (100.00)	0	0
Total (*n* = 51)	2 (3.92)	6 (11.76)	0	1 (1.96)	0	4 (7.84)	40 (78.43)	9 (17.65)	35 (68.63)	0	8 (15.69)

Among turkey strains *tet M* gene was detected in 40 (78.43%) isolates, whereas gene *ermB* was found in 35 (68.63%) *Enterococcus* spp. Only four strains (7.84%) were found to encode the *tetO* gene. *vanC-1* gene was found in all *E. gallinarum* strains investigated that were isolated from humans and turkeys.

Comparing the presence of *tet* genes and resistance to tetracycline among human strains, 42 (87.5%) and 24 (50%) tetracycline-resistant *Enterococcus* spp. possessed the *tetM* and *tetO* genes, respectively (Supplementary Table S1). In 12 (57.14%) *Enterococcus* isolated from humans that were resistant to erythromycin, *ermB* gene was found. Moreover, eight human strains (27.59%) intermediately susceptible to erythromycin were found to encode the *ermB* gene. Among 14 human isolates resistant to vancomycin, *vanA* gene was found only in one strain (7.14%), whereas *vanC-1* gene was present in five isolates (35.71%). In addition, one intermediately susceptible strain had a *vanC-1* gene (5.26%). Three or more genes, in one isolate, coding resistance to different antimicrobials were found in 23 (41.07%) *Enterococcus* spp. obtained from humans, as follows: 16 strains of *E. faecalis* (69.57%), 5 of *E. gallinarum* (21.74%), and 2 isolates of *E. faecium* (8.69%).

Among turkey strains resistant to tetracycline, *tetM* gene was found in 39 (81.25%) isolates, whereas *tetO* gene occurred only in four *Enterococcus* strains (8.33%) (Supplementary Table S2). *ErmB* and *ermA* genes were found in 32 (82.05%) and 9 (23.08%) turkey strains resistant to erythromycin, respectively. In addition, the *ermB* gene was detected in three turkey strains (42.86%) intermediately susceptible to erythromycin. Among four erythromycin-resistant turkey isolates (10.26%), it was not possible to detect *ermB* gene; among these strains the presence of *ermA* gene was observed. The presence of *vanA* and *vanC-1* genes was confirmed in four turkey strains (22.22%) intermediately susceptible to vancomycin. In 17 (33.33%) turkey isolates the presence of 3 or more resistance genes was found, as follows: *E. faecalis* (70.59%), *E. faecium* (23.53%), and *E. gallinarum* (5.88%).

The aminoglycoside resistance gene (*aac(6’)Ie-aph(2”)Ia*) was detected in 13 *E. faecalis* strains (23.21%) isolated from humans and in 8 *Enterococcus* spp. (15.69%) obtained from turkeys (4 strains of *E. faecalis* and 4 strains of *E. faecium*). All these isolates were susceptible to gentamicin.

### Biofilm formation

Among the human and turkey strains studied, all isolates had the ability to form biofilms, but at different levels. After 24 hours of incubation, in 11 (no. 125, 131, 190, 191, 196, 979, 982, 1872, 1963, 2006, 179B) of 56 human strains a medium positive ability of biofilm formation was observed (Supplementary Table S1). Forty-five (80.36%) *Enterococcus* isolated from humans formed strong biofilm, whereas after 48-hour incubation 100% of human strains produced strong biofilm. Among human strains, the bacteria with no possibility of biofilm formation were not detected.

Turkey strains were distinguished by stronger biofilm production after a 24-hour incubation (98.04% of strains) in comparison with human isolates, which was statistically significant (*p* = 0.019). In 3 of 51 *Enterococcus* strains (no. Z2, JR6, and R1), a medium positive ability to biofilm formation after 24 hours of incubation was observed, whereas after 48 hours of incubation the percentage of strains producing strong biofilm was 100% (Supplementary Table S2).

There was a statistically significant difference (*p* ≤ 0.05) in the OD value for biofilm formed after 24 and 48 hours of incubation, in both *Enterococcus* strains isolated from humans and turkeys.

In our results, a predominance of *E. faecalis* over other species was not observed. All species examined formed a biofilm at the same level.

## Discussion

Bacteria of the genus *Enterococcus* are found in the digestive tract of human beings and animals, forming part of the physiological flora. Epidemiological research on *Enterococcus* spp. indicates that together with feces, these bacteria end up in the environment that, thanks to their high adaptability to various environments, easily colonizes them.^42^ Hence, their widespread occurrence in soil, water, and sewage and on plants and fruits. In this way, they contaminate raw materials of both animal and vegetable origin.^43^

In this study, antimicrobial resistance, the presence of selected resistance genes, and biofilm formation ability of *Enterococcus* strains isolated from humans and turkeys were compared. Our study revealed that *E. faecalis* and *E. faecium* were the most prevalent enterococcal species isolated from humans and turkeys, similar to results obtained by other authors.^15,44–46^

In this study, the highest percentage of resistance among human and turkey strains was against tetracycline and erythromycin, which is similar to results published by other authors.^46–48^ Both these antimicrobial groups are often used in veterinary and human medicine, especially regarding enteric infections. Aarestrup *et al.*^15^ reported that 74% of *E. faecium* and 44% of *E. faecalis* strains isolated from broilers were resistant to erythromycin. The resistance to tetracycline among these isolates was as follows: 59% of *E. faecalis* and 32% of *E. faecium*. A high prevalence of resistance to erythromycin and tetracycline in *Enterococcus* spp. isolated from retail meats in Canada was reported by Aslam *et al.*,^44^ where 90% and 28% of *E*. *faecalis* isolated from turkey meat were resistant to tetracycline and erythromycin, respectively. Another study on multiple-antibiotic resistance of *E. faecalis* and *E. faecium* was conducted in Canada by Tremblay *et al.*^49^ The isolates were collected from cecal contents in broiler chickens and turkeys. The percentages of *E. faecalis* and *E. faecium* resistant to erythromycin were 72.6 and 80.3, respectively. A similar observation was indicated in our results with reference to *Enterococcus* strains isolated from turkeys. There is a hypothesis that unregulated use of antimicrobial agents, in food-animal production, has led to the emergence and spread of antibiotic resistance, among *Enterococcus* spp.^50^ The prevalence of resistant strains is very low in countries where the use of antibiotics in poultry industry is uncommon.^51^

Among strains obtained from turkeys, only 15.69% of *Enterococcus* spp. were resistant to vancomycin, whereas up to 25% of human isolates were resistant to this antibiotic. This difference in the level of resistance may be because of the frequent use of vancomycin in human medicine, whereas the use of avoparcin was banned in 1997. Antimicrobial resistance of *Enterococcus* isolated from humans was analyzed in 2000–2015, in Turkey, by Kilbas and Ciftci.^52^ The results demonstrated that the mean resistance rate of *E. faecalis* to vancomycin was 1.0–2.2%, whereas the mean resistance rate of *E. faecium* to vancomycin was 10.3–11.3%. Various authors report a broad range of prevalence of vancomycin-resistant strains, isolated from poultry or the poultry environment, from 0% to 94%.^53–55^ The results obtained by Borgen *et al.*^56^ suggested that vancomycin-resistant enterococci (VRE) may persist subsequently in the compartments and colonize the next batch of broilers. Nilsson *et al.*^54^ revealed that even the low degree of VRE contamination in a broiler farm was sufficient for amplification and spread of antibiotic-resistant bacteria in the environment.

In this study, high-level resistance to gentamicin among all isolates investigated was not observed, which is in agreement with results obtained by other authors.^57^

The results from this study documented that 18 of 56 strains (32.14%) isolated from humans and 22 of 51 strains (43.14%) isolated from turkeys were resistant to three or more antimicrobials. Authors from Germany, Maasjost *et al.*^53^ observed that 89 of 145 *Enterococcus* isolates (61.38%) were resistant to three or more antimicrobial agents, which is in agreement with our results. The presence of MDR enterococci in turkey flocks may represent a hazard for public health, considering the contact humans have with poultry products contaminated with these bacteria. It has been proven that *E. faecalis* of human and poultry origin share virulence genes, supporting the zoonotic potential of *E. faecalis.*^12^

In this study, the most common isolated resistance gene was *tetM*, which was found in 48 (85.71%) and 40 (78.43%) of human and turkey strains, respectively. In addition, 39 (81.25%) of 48 turkey strains resistant to tetracycline were found to encode the *tetM* gene. Aarestrup *et al.*^15^ reported that 92% and 94% of *E. faecalis* and *E. faecium* strains, respectively, isolated from broilers that were resistant to tetracycline were found to encode *tetM* gene. Similar results were obtained by Aslam *et al.*,^44^ who found *tetM* gene in 89% of *E. faecalis* isolates from chickens and in 84% of those from turkeys. *Enterococcus* spp. are known to acquire and transfer antibiotic resistance genes easily, especially those located on mobile genetic elements (transfer of plasmids and transposons, chromosomal exchange, mutations), to other potentially pathogenic bacteria in the chicken intestine.^12,58,59^ Therefore, enterococci are regarded as a potential source for the spread of resistance genes among bacteria.

High-level gentamicin resistance is associated with bifunctional *aac6’-aph2”* aminoglycoside-modifying enzymes, which also confers high-level resistance to amikacin, tobramycin, kanamycin, netilmicin, and dibekacin, with the exception of streptomycin.^60^ In our study, the presence of the *aac(6’)Ie-aph(2”)Ia* gene was detected in 23.21% and 15.69% of *Enterococcus* strains isolated from humans and turkeys, respectively. It was surprising to demonstrate the presence of this gene being susceptible to gentamicin strains. These findings may indicate that the presence the *aac(6’)Ie-aph(2”)Ia* gene may be related to the widespread use of aminoglycosides in human medicine and aminocyclitols, including spectinomycin, in veterinary medicine,^61^ or that the gene was inactive, or were not being expressed.^62^

In this study, the *ermB* gene was the most frequently observed among erythromycin-resistant isolates obtained from humans (42.86%) and turkeys (68.63%). This is consistent with results of other researchers, in which the most common gene encoding macrolide resistance in enterococci was *ermB.*^15^

All the isolates used in this study had the ability to form a biofilm. Among human and poultry strains, the bacteria were characterized by a continuous increasing biofilm production along with incubation time. One study in Italy found that 80% of *E. faecalis* in human isolates and 48% of *E. faecium* strains had formed biofilms, whereas another study conducted by these authors on 47 human isolates showed a biofilm phenotype associated with 87% of *E. faecalis,* and only 16% of *E. faecium* strains.^63^ A study conducted in Spain showed that more than half of the 152 clinical isolates of *E. faecalis* were able to form biofilms *in vitro* without the biofilm phenotype exhibited by *E. faecium*, *E. gallinarum*, or *Enterococcus avium* strains.^64^ Research conducted in Poland showed that higher biofilm formation ability was also found among *E. faecalis* isolates, but not from four other species of enterococci tested.^65^ A study of 171 enterococcal clinical isolates at a hospital in India showed that about a quarter of *E. faecalis* strains (*n* = 44) were able to form biofilms *in vitro*, unlike any of the isolated *E. faecium* strains.^66^ These studies indicate that *E. faecalis* is an important bacterium that produces biofilms in intestinal infections, which somewhat limits the therapeutic options of antibiotics used to treat such infections.

Oliveira^67^ investigated biofilm formation in *Enterococcus* spp. among broilers from intensive and extensive farms. The purpose of the study was to determine whether the breeding method has an impact on the development and intensity of biofilm among poultry populations. The authors noticed that the biofilm was formed more quickly and intensively in intensive than in extensive cultures. After 24 hours of incubation, the biofilm formed 27.8% of the strains from extensive breeding and 68.8% of intensive breeding strains. After 48-hour incubation the percentage increased to 38.9% for extensive breeding and up to 75% for intensive breeding. The results presented indicate similarity of strains from intensive culture with strains analyzed in this study—strains created a very strong biofilm after the first 24 hours of incubation time.

Different results were obtained by Necidová^23^ who studied the formation of biofilm among *E. faecium* and *E. faecalis* strains isolated from food. The samples came from milk and dairy products obtained on the farm in the Czech Republic. The obtained results indicated that the biofilm was more often formed by *E. faecalis.* Among the surveyed population, only 28% of strains formed a biofilm. In our research, the predominance of a specific *Enterococcus* species in biofilm formation has not been noticed—all examined species created a biofilm at the same level. This may indicate that the subjects have strains of virulence factor, which is the enterococcal surface protein (Esp) that promotes colonization at different surfaces and biofilm formation.^68^

As indicated by the results mentioned previously, the formation of biofilm by strains of *Enterococcus* spp., both isolated from humans and turkeys, may be an additional factor that increases the pathogenic potential of enterococci. High antimicrobial resistance combined with the ability to create a biofilm locates the genus *Enterococcus* as one of the most dangerous pathogens that could pose risk to health and life. That is why it is important to study and control the population of these microorganisms in different groups, both in animals and people to prevent serious development of epidemics in hospital environments or mass losses among farm animals.

In our study, all *Enterococcus* species examined formed a biofilm at the same level. This may indicate that the strains tested had a virulence factor, Esp, which promotes the colonization of various surfaces and the formation of biofilms. *Enterococcus* spp. have the ability to form a biofilm and survive in phagocytic cell.^69^ Bacterial cells that are an integral part of biofilm are much more resistant to bactericides than plankton forms. A biofilm can function under conditions in which the survival of single cells would be difficult, and in many cases even impossible.^26^

## Conclusions

Enterococci belonging to human and animal gastrointestinal flora are widely distributed in the environment. They are opportunistic bacteria that can cause severe infections, with the ability to acquire, express, and transfer antimicrobial resistance. This study showed frequent occurrence of antimicrobial resistance, especially to tetracycline and erythromycin, in *E. faecalis* and *E. faecium* isolated from humans and turkeys.

The significant usage of tetracycline antibiotics in poultry production and human medicine in Poland has led, as shown in our study, to the emergence of tetracycline-resistant *Enterococcus* strains. Moreover, results presented in this study indicate that the intestinal enterococci of healthy turkeys, which can contaminate poultry meat, could be a reservoir of *blaZ, vanA*, *vanC-1, tetO, tetM, ermA, ermB*, and *aac(6’)Ie-aph(2”)Ia* genes.

Monitoring antimicrobial resistance to *Enterococcus* and the appropriate use of antimicrobials in animal food production are essential for decreasing drug resistance in bacterial pathogens.

## Supplementary Material

Supplemental data

Supplemental data
