# Supplementary material for: Antimicrobial Resistance and Biofilm Formation in Enterococcus spp. Isolated from Humans and Turkeys in Poland
Source: Microb Drug Resist. 2019 Mar 8;25(2):277–86. doi: 10.1089/mdr.2018.0221 (PMC6441282; doi:10.1089/mdr.2018.0221)
Supplement: Supplemental data [file Supp_Table1.pdf]

Supplementary Data

Supplementary Table S1. Antimicrobial Susceptibility and Presence of Resistance Genes in *Enterococcus* spp. Isolated from Humans (n=56)

		Antimicrobials							*Resistance genes*											*Biofilm formation*	
No. of strain	*Species*	*AMP*	*AMX*	*VAN*	*CIP*	*TET*	*ERY*	*GEN*	*blaZ*	*vanA*	*vanB*	*vanC*	*tetK*	*tetO*	*tetM*	*ermA*	*ermB*	*ermC*	*aac(6')Ie-aph(2'')Ia*	*24 h*	*48 h*
25	*Enterococcus faecalis*	S	S	R	R	R	I	S						+	+		+		+	Strong	Strong
35	*E. faecalis*	S	S	R	R	R	I	S							+					Strong	Strong
39	*E. faecalis*	R	R	R	R	R	I	S											+	Strong	Strong
44	*E. faecalis*	S	S	I	S	I	R	S						+						Strong	Strong
45	*E. faecalis*	R	S	I	R	R	R	S							+				+	Strong	Strong
73	*E. gallinarum*	R	S	R	R	R	I	S				+		+	+		+			Strong	Strong
77	*E. gallinarum*	S	S	R	I	R	I	S				+		+	+					Strong	Strong
80	*E. faecalis*	S	S	S	R	R	R	S						+	+					Strong	Strong
81	*E. faecalis*	S	S	R	I	R	I	S							+		+			Strong	Strong
83	*E. gallinarum*	S	S	R	I	R	I	S		+		+			+		+			Strong	Strong
90	*E. faecalis*	S	S	S	I	R	I	S				+			+					Strong	Strong
103	*E. faecalis*	S	S	I	I	R	I	S							+					Strong	Strong
121	*E. faecalis*	S	S	S	I	R	R	S									+			Strong	Strong
123	*E. faecalis*	S	S	I	R	R	R	S						+	+					Strong	Strong
125	*E. faecalis*	R	R	R	R	R	R	S							+		+		+	*Medium*	Strong
130	*E. faecalis*	R	R	S	R	R	R	S						+			+		+	Strong	Strong
131	*E. faecalis*	S	S	I	I	I	I	S						+	+				+	*Medium*	Strong
145	*E. faecalis*	S	S	S	I	R	I	S							+		+			Strong	Strong
150	*E. faecalis*	R	S	S	R	R	R	S							+		+			Strong	Strong
171	*E. faecalis*	S	S	S	I	R	R	S						+	+		+			Strong	Strong
172	*E. faecium*	S	S	I	R	I	I	S						+						Strong	Strong
173	*E. faecalis*	S	S	S	I	R	I	S						+	+					Strong	Strong
178	*E. faecium*	S	S	S	I	R	I	S						+	+					Strong	Strong
180	*E. faecalis*	S	S	S	I	R	I	S												Strong	Strong
184	*E. faecalis*	S	S	S	I	R	S	S						+	+		+			Strong	Strong
190	*E. faecalis*	S	S	S	R	S	R	S						+	+		+			*Medium*	Strong
191	*E. faecalis*	S	S	S	R	R	R	S						+	+					*Medium*	Strong
192	*E. faecalis*	S	S	S	I	R	I	S						+	+					Strong	Strong
196	*E. faecalis*	S	S	S	I	S	I	S						+	+		+			*Medium*	Strong
979	*E. faecalis*	S	S	R	S	R	I	S												*Medium*	Strong
982	*E. faecalis*	S	S	R	I	R	I	S												*Medium*	Strong
989	*E. faecalis*	S	I	S	I	R	S	S							+					Strong	Strong
1003	*E. faecalis*	S	S	S	I	R	I	S						+	+				+	Strong	Strong
1010	*E. faecalis*	S	S	I	I	R	I	S						+	+		+		+	Strong	Strong

(*continued*)

Supplementary Table S1. (Continued)

No. of strain	Species	*Antimicrobials*							*Resistance genes*											*Biofilm formation*	
		AMP	*AMX*	*VAN*	*CIP*	*TET*	*ERY*	*GEN*	*blaZ*	*vanA*	*vanB*	*vanC*	*tetK*	*tetO*	*tetM*	*ermA*	*ermB*	*ermC*	*aac(6')Ie-aph(2'')Ia*	*24 h*	*48 h*
1013	*E. faecalis*	S	S	I	S	R	S	S						+	+	+			+	Strong	Strong
1021	*E. faecalis*	S	S	I	R	R	R	S							+		+			Strong	Strong
1022	*E. faecalis*	S	S	I	I	R	S	S						+	+		+			Strong	Strong
1130	*E. faecalis*	S	S	S	I	R	R	S						+	+		+			Strong	Strong
1131	*E. faecalis*	S	S	I	I	R	I	S							+				+	Strong	Strong
1134	*E. faecalis*	S	S	I	S	R	I	S							+					Strong	Strong
1135	*E. faecalis*	S	S	I	S	S	I	S							+					Strong	Strong
1841	*E. faecalis*	S	S	I	S	R	I	S						+	+		+			Strong	Strong
1868	*E. gallinarium*	S	S	R	I	R	R	S				+		+	+					Strong	Strong
1872	*E. faecium*	S	S	R	I	R	R	S						+	+		+			*Medium*	Strong
1873	*E. gallinarium*	S	S	R	I	R	R	S				+		+	+		+			Strong	Strong
1931	*E. faecalis*	S	S	R	S	R	R	S							+		+			Strong	Strong
1949	*E. faecium*	S	S	I	S	R	I	S						+	+					Strong	Strong
1963	*E. gallinarum*	S	S	I	R	R	I	S				+			+					*Medium*	Strong
1983	*E. faecalis*	S	S	I	I	R	S	S							+		+		+	Strong	Strong
2000	*E. faecalis*	S	S	I	S	R	S	S						+	+	+	+			Strong	Strong
2002	*E. gallinarum*	S	S	S	I	S	I	S						+	+					Strong	Strong
2006	*E. faecium*	S	S	S	S	R	R	S						+	+		+			*Medium*	Strong
133A	*E. faecalis*	R	R	S	R	R	R	S							+				+	Strong	Strong
133B	*E. faecalis*	R	R	I	R	R	R	S							+				+	Strong	Strong
179A	*E. faecalis*	S	S	S	S	R	I	S							+					Strong	Strong
179B	*E. faecalis*	S	S	S	S	S	R	S							+					*Medium*	Strong

AMP, ampicillin (10 μg); AMX, amoxicillin/clavulanic acid (20/10 μg); VAN, vancomycin (30 μg); CIP, ciprofloxacin (5 μg); TET, tetracycline (30 μg); ERY, erythromycin (15 μg); GEN, gentamicin (120 μg).

S, susceptible; I, intermediate; R, resistant; (+) - positive result, presence of the gene.
