# Supplementary material for: Antimicrobial Resistance and Biofilm Formation in Enterococcus spp. Isolated from Humans and Turkeys in Poland
Source: Microb Drug Resist. 2019 Mar 8;25(2):277–86. doi: 10.1089/mdr.2018.0221 (PMC6441282; doi:10.1089/mdr.2018.0221)
Supplement: Supplemental data [file Supp_Table2.pdf]

Supplementary Table S2. Antimicrobial Susceptibility and Presence of Resistance Genes in *Enterococcus* spp. Isolated from Turkey (n=51)

		Antimicrobials							*Resistance genes*											*Biofilm formation*	
No. of strain	*Species*	*AMP*	*AMX*	*VAN*	*CIP*	*TET*	*ERY*	*GEN*	*blaZ*	*vanA*	*vanB*	*vanC*	*tetK*	*tetO*	*tetM*	*ermA*	*ermB*	*ermC*	*aac(6')Ie-aph(2'')Ia*	*24 h*	*48 h*
Z1	*E. faecalis*	S	S	S	R	R	R	S		+							+			Strong	Strong
Z2	*E. faecalis*	S	S	S	I	R	R	S							+					*Medium*	Strong
Z3	*E. faecalis*	S	S	S	I	R	R	S							+		+			Strong	Strong
JK1	*E. faecalis*	S	S	S	I	R	R	S							+					Strong	Strong
JK2	*E. faecium*	S	S	I	R	R	R	S		+				+			+			Strong	Strong
JK3	*E. faecalis*	S	S	R	I	R	R	S							+		+			Strong	Strong
JK4	*E. faecalis*	S	S	S	I	R	R	S		+							+			Strong	Strong
JB1	*E. faecalis*	S	S	I	R	R	R	S		+					+		+			Strong	Strong
JB2	*E. faecalis*	R	R	R	R	R	R	S							+		+		+	Strong	Strong
JB3	*E. faecalis*	R	R	R	R	R	R	S							+		+			Strong	Strong
JB4	*E. faecium*	R	R	I	I	R	R	S							+		+			Strong	Strong
JB5	*E. faecium*	R	R	I	R	R	R	S									+		+	Strong	Strong
JB6	*E. faecalis*	R	R	I	I	R	R	S		+					+		+			Strong	Strong
JB7	*E. faecalis*	R	R	S	I	R	R	S	+											Strong	Strong
JR1	*E. faecalis*	S	S	S	I	R	I	S							+					Strong	Strong
JR2	*E. faecalis*	S	S	S	I	R	S	S		+					+				+	Strong	Strong
JR3	*E. faecalis*	S	S	S	I	R	S	S												Strong	Strong
JK4	*E. faecalis*	I	I	S	I	R	R	S						+	+		+		+	Strong	Strong
JR5	*E. faecalis*	S	S	S	I	R	R	S							+					Strong	Strong
JR6	*E. faecalis*	S	S	S	S	R	S	S							+					*Medium*	Strong
JR7	*E. faecalis*	S	S	S	I	R	S	S	+											Strong	Strong
R1	*E. faecalis*	S	S	S	S	R	R	S						+	+		+			*Medium*	Strong
R2	*E. faecalis*	S	S	S	S	R	I	S						+	+		+			Strong	Strong
R3	*E. faecalis*	S	S	S	S	R	I	S							+		+			Strong	Strong
R4	*E. faecalis*	S	S	I	S	R	R	S							+		+			Strong	Strong
M1	*E. faecium*	R	I	S	R	R	R	S							+		+			Strong	Strong
M2	*E. faecalis*	S	S	I	S	R	R	S							+	+	+		+	Strong	Strong
M3	*E. faecalis*	R	I	S	R	R	R	S							+	+	+			Strong	Strong
M4	*E. faecalis*	S	S	R	S	R	R	S							+		+			Strong	Strong
M5	*E. faecium*	R	R	R	R	R	R	S							+	+	+		+	Strong	Strong

(*continued*)

Supplementary Table S2. (Continued)

		Antimicrobials							Resistance genes											Biofilm formation	
No. of strain	*Species*	*AMP*	*AMX*	*VAN*	*CIP*	*TET*	*ERY*	*GEN*	*blaZ*	*vanA*	*vanB*	*vanC*	*tetK*	*tetO*	*tetM*	*ermA*	*ermB*	*ermC*	*aac(6')Ie-aph(2'')Ia*	*24 h*	*48 h*
M6	*E. faecium*	R	R	I	R	R	R	S							+	+				Strong	Strong
M7	*E. faecium*	R	R	R	R	R	R	S							+	+	+		+	Strong	Strong
MS1	*E. faecalis*	S	S	I	S	R	I	S							+					Strong	Strong
MS2	*E. faecalis*	S	S	S	S	R	R	S							+	+	+			Strong	Strong
MS3	*E. faecalis*	S	S	I	S	R	R	S							+		+			Strong	Strong
MS4	*E. faecalis*	S	S	I	S	R	R	S							+		+			Strong	Strong
MS5	*E. faecalis*	S	S	I	S	R	I	S							+		+			Strong	Strong
MS6	*E. faecalis*	S	S	R	S	R	R	S							+	+	+			Strong	Strong
MS7	*E. faecalis*	S	S	S	S	R	R	S							+					Strong	Strong
JM1	*E. faecium*	S	S	S	I	R	R	S							+					Strong	Strong
JM2	*E. faecium*	R	R	S	R	R	R	S							+		+		+	Strong	Strong
JM3	*E. faecalis*	S	S	I	S	R	R	S							+		+			Strong	Strong
JM4	*E. faecalis*	S	S	I	S	R	S	S							+					Strong	Strong
JM5	*E. faecalis*	S	S	I	S	S	R	S							+		+			Strong	Strong
JM6	*E. faecalis*	R	R	S	I	R	R	S							+	+	+			Strong	Strong
JM7	*E. faecalis*	R	R	R	I	R	R	S							+		+			Strong	Strong
JZ1	*E. faecalis*	S	S	S	I	S	I	S												Strong	Strong
JZ2	*E. faecalis*	S	S	I	S	R	R	S									+			Strong	Strong
JZ3	*E. faecalis*	S	S	S	I	R	I	S												Strong	Strong
JZ4	*E. faecalis*	R	R	I	S	S	R	S									+			Strong	Strong
JZ5	*E. gallinarium*	R	R	I	S	R	R	S				+			+	+	+			Strong	Strong

AMP, ampicillin (10 μg); AMX, amoxicillin/clavulanic acid (20/10 μg); VAN, vancomycin (30 μg); CIP, ciprofloxacin (5 μg); TET, tetracycline (30 μg); ERY, erythromycin (15 μg); GEN, gentamicin (120 μg).

S, susceptible; I, intermediate.
